# Role of calcination process of natural colemanite powder on compressive strength property of concrete

**DOI:** 10.1016/j.heliyon.2024.e41264

**Published:** 2024-12-17

**Authors:** Nihat Utku Guner, Sezai Kutuk, Tuba Kutuk-Sert

**Affiliations:** a Department of Civil Engineering, Faculty of Engineering and Architecture, Recep Tayyip Erdogan University, 53100, Rize, Turkey; b Department of Marine Engineering, Faculty of Turgut Kiran Maritime, Recep Tayyip Erdogan University, 53900, Derepazari, Rize, Turkey

**Keywords:** Boron, Mineral additive, Calcination, Concrete, Compressive strength, CO_2_ emissions

## Abstract

The use of boron minerals as an additive is important in terms of reducing CO_2_ emissions and providing input to the economy. Sustainable natural colemanite was subjected to calcination at 550 °C in order to concentrate the amount of B_2_O_3_. For the characterization of calcined mineral, XRD, TGA/DTA, and B_2_O_3_ component tests were carried out. It was observed that the structure of natural colemanite changed, and the B_2_O_3_ value heightened by 11 %. Then, natural and calcined minerals were added to the concrete mixture in proportions of 1.25 %, 2.5 %, 5 %, 7.5 %, 10 %, 12.5 %, and 15 % by weight. Ultrasonic pulse velocity, Schmidt hardness, and compressive strength tests were fulfilled. In the samples with natural additives, the lowest compressive strength was 32 MPa for the reference sample, whereas the highest strength was 44 MPa for the 10 % natural colemanite sample. In other words, the compressive strength increased by 39 %. In the samples with calcined additives, the highest compressive strength was 30 MPa for the 2.5 % calcined colemanite sample; that is, it improved by 24 %. It is realized that the cement can be saved since high strength can be obtained in concrete with natural and calcined colemanite additives.

## Highlights

• With calcination, B_2_O_3_ content of colemanite powder increased by 11 %.
• The major phase of the crystal structure was transformed into calcite.
• 71 % of the crystal water was removed from the structure.
• The compressive strength of the concrete sample improved by 24 %.
• Higher compressive strength was obtained with a lower additive ratio.

## Graphical abstract

**Figure:**
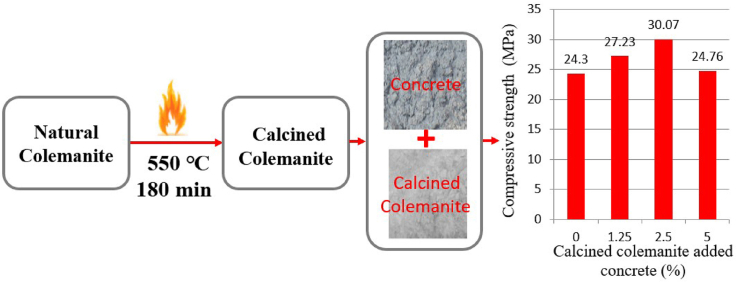

## 1 Introduction

The cement industry ranks third in energy consumption with 7 %, unfortunately it is located in second in CO_2_ emissions with 7 %. Owing to the growth of the world's population and high-speed urbanization, it is expected that there will be an increment of 12 %–23 % in cement consumption and also a rise of 4 % in CO_2_ emissions by 2050 [1]. Therefore, researchers have been doing important studies in recent years to decrease the dependence on cement and CO_2_ emissions. In many studies, mineral additives used in place of cement reduce the heat of hydration [2], diminish the carbon dioxide emitted into the atmosphere by approximately 25 %–30 % [3], improve freeze-thaw cycles and sulfate resistance of concrete [4], reduce the negative effects of cold jointed concrete [5], increase the resistance to chloride-induced corrosion in concrete [6], and considerably change other strength properties of concrete [7,8]. In addition, mineral additives have environmental and economic benefits for cement concrete [[9], [10], [11]].

Boron minerals contain B_2_O_3_ (precious component) in their structure at different ratios. Although there are more than 230 types, tincal (Na_2_O·2B_2_O_3_·10H_2_O), ulexite (Na_2_O·2CaO·5B_2_O_3_·16H_2_O) as well as colemanite (2CaO·3B_2_O_3_·5H_2_O) types are the most important industrial boron minerals. The West Anatolia region, having 73 % of the world's deposits of boron as B_2_O_3_ with 949 million tons and 56 % of the world trade, has a very important place in this field. Therefore, R&D studies are rapidly increasing day by day in order to extract, process, and use boron minerals most efficiently. Colemanite is one of the most extracted minerals in Turkey for a raw material. This mineral is used in the industry in its raw form for some materials and in its processed form for some materials. The usage areas of colemanite are briefly as follows: disinfectant, detergent, fertilizer, nuclear power station, fire retardant, textile, glass, glass fiber, metallurgical clinker, enamel, cement, road concrete, asphalt concrete, and more [12,13].

There are a few studies related to the fact that it is possible to increase the amount of B_2_O_3_ in the natural colemanite mineral thanks to the calcination process [[14], [15], [16]]. The advantage of obtaining larger B_2_O_3_ is to increase the purity percentage in colemanite ore and use it in economically valuable products. However, conditions such as the natural form of the colemanite mineral, B_2_O_3_ amount, impurity components, impurity amount, particle size, calcination temperature, calcination time, and so on change the results in application areas. Sener et al. [14] examined the TG data by calcining the natural colemanite mineral (−10 mm, B_2_O_3_: 49.32 wt%). In the TG curve of the natural colemanite mineral, they determined that sudden dehydration occurred between 390 °C and 399 °C and the mass loss decreased to 45 % at 500 °C. Later, they mixed natural colemanite and natural ulexite minerals. It was deduced that the B_2_O_3_ amount of the mixtures calcined at different temperatures and times increased, but was not the same. Celik and Suner [15] performed the thermodynamic analysis of 14 minerals with hydrated boron. They reported that colemanite mineral (an assay purity of >98 %) released all 5 mol of crystalline water between 262 °C and 412 °C. This was attributed to the high enthalpy value and the high onset of dehydration temperature. Yildiz [16] calcined the natural colemanite mineral (−250 μm, B_2_O_3_: 42 wt%) at 600 °C and found that the amount of B_2_O_3_ reached to 53 wt%.

In recent decades, numerous researchers from various countries have been working extensively on ground materials. This is because their structural, magnetic, electrical, thermal, and mechanical characteristics are stronger than unground materials. Kutuk-Sert [17] added ground natural colemanite minerals (−75 μm, −45 μm, and −25 μm) as an additive to the concrete road. At the study's initial step, colemanite minerals were analyzed. The best average particle size was quantified with a laser particle size device as 8.111 μm for the −25 μm mineral. Likewise, the minimum particle size was detected as 316 nm for the −25 μm mineral. Thus, it was found that the colemanite mineral particle size was satisfactorily decreased to the submicron scale. Besides, findings from the laser particle size device were verified with scanning electron microscope (SEM) micrographs. At the study's final step, the −25 μm mineral was mixed as an additive to the concrete road. As a result, it was determined that it covered the spaces between the aggregates, resulting in strong adhesion. Hence, a gain in the concrete's compressive strength was obtained. In a study examining the chemical component and crystal structure characteristics of −75 μm, −45 μm, and −25 μm colemanite minerals [18], it was concluded that the B_2_O_3_ component did not alter considerably with the grinding and sieving procedures. More notably, the crystallite size of colemanite was effectively dropped by 63.6 nm to the nanoscale.

Various methods are used to determine the concrete quality. These methods are divided into two, destructive testing and non-destructive testing. The most applied destructive tests are compressive, flexural tensile, and splitting tensile strength. Nevertheless, the most widely applied non-destructive tests are ultrasonic pulse velocity (UPV) and Schmidt hardness. Non-destructive testing methods are highly preferred because of their advantages such as not damaging the sample, ease of application, minimizing time loss, and being economical. Kutuk [17] added natural colemanite minerals (B_2_O_3_: 40 wt%) with sizes of −45 μm and −75 μm to the concrete samples. For concrete samples with −45 μm colemanite, the 28 days compressive strength heightened from 35 to 39 MPa up to 2 wt% additive. For concrete samples with −75 μm colemanite, the compressive strength gradually improved from 35 to 42 MPa up to 5 wt% additive.

In a study investigating the effect of boron wastes as a filler aggregate in hot mix asphalt pavement [19], it was found that the asphalt mixture produced with boron waste was suitable for use. According to the Highways Technical Specification in Turkey, it was recommended to utilize it as a binding layer, especially in regions with hot climates. For the purpose of utilizing boron minerals in road construction, Keskin et al. [20] mixed borax pentahydrate (−1.18 mm, B_2_O_3_: 47.90 wt%), crushed boron waste (−1.18 mm, B_2_O_3_: 11.84 wt%), and anhydrous borax (−1.18 mm, B_2_O_3_: 69.30 wt%) materials into asphalt concrete samples. Marshall design and creep tests were applied to the asphalt samples and then the measurements showed that the three materials were in compliance with the specifications. In another study [21], ground natural colemanite minerals (−25 μm, B_2_O_3_: 30 wt%) were added to asphalt mixtures at 5 % and 10 % ratios. It was decided that the mechanical properties of warm mix asphalt such as the rutting resistance and vertical deformation were improved with ground colemanite mineral.

### 1.1 Research Significance

Although the colemanite mineral has been added to concrete/asphalt samples as an additive for construction material in the literature, a calcined colemanite mineral has not been added to concrete samples and its role in compressive strength has not been examined. The use of boron mineral, which has abundant reserves in the world, is important in terms of reducing CO_2_ emissions and providing input to the economy. Therefore, the purpose of the present study is to apply the calcination process to the natural sustainable colemanite mineral and to interpret its mechanical effects on concrete samples/pavements.

## 2 Material and methods

### 2.1 Preparation and characterization of calcined colemanite mineral

#### 2.1.1 Initial material

The ground natural colemanite mineral was obtained from the Eti Maden Bigadic Boron Operation Directorate. The colemanite product, which was sieved with 45 μm mesh, was utilized as the starting material and was called NC for convenience. Bravais lattice of colemanite mineral is monoclinic, its specific gravity is 2.42 g/cm^3^, its Mohs hardness is 4–4.5, and its solubility in water is slow. Its chemical component values are listed in Table 1 [22].

**Table 1:** Chemical composition analyses of nature colemanite.

Component	(wt. %)
B_2_O_3_	40.00 ± 0.50
CaO	27.00 ± 1.00
SiO_2_	4.00–6.50
MgO	3.00 max
SrO	1.50 max
SO_4_	0.60 max
Al_2_O_3_	0.40 max
Na_2_O	0.35 max
Fe_2_O_3_	0.08 max
As	35 ppm max
LOI	24.60 max
Humidity	1.00 max

**Fig. 1:**
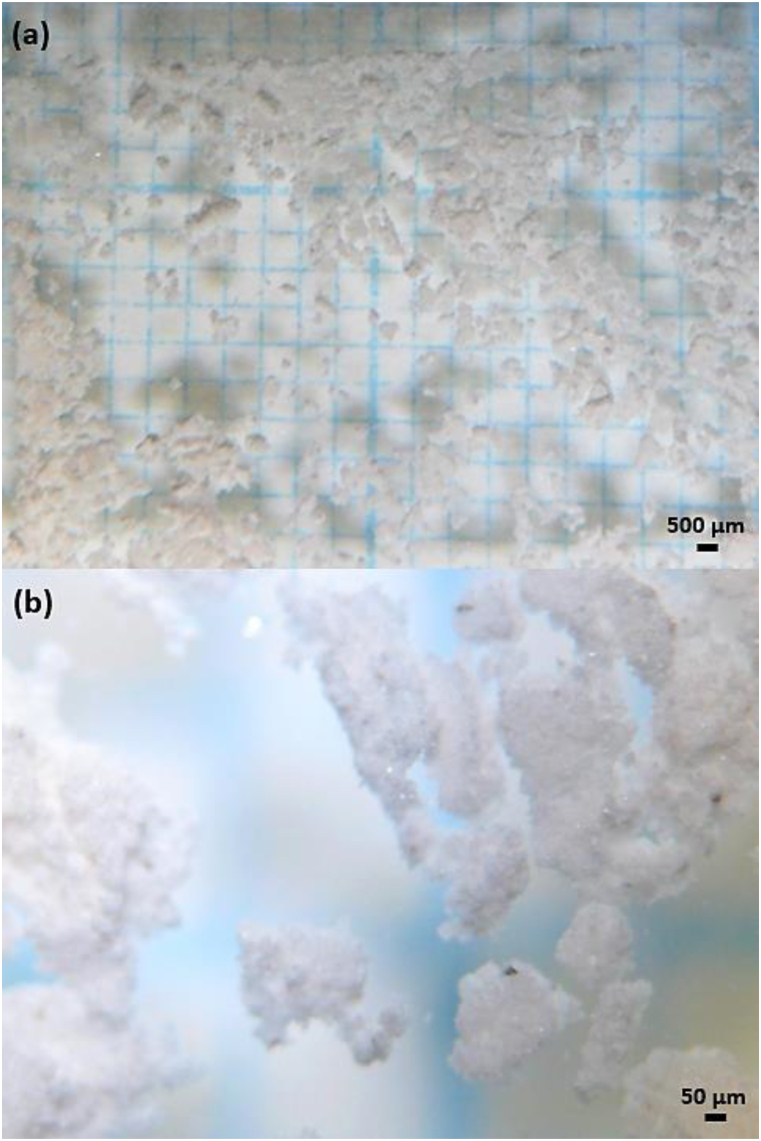
The SOM images of natural colemanite under (a) × 6 and (b) × 45 magnification.

**Fig. 2:**
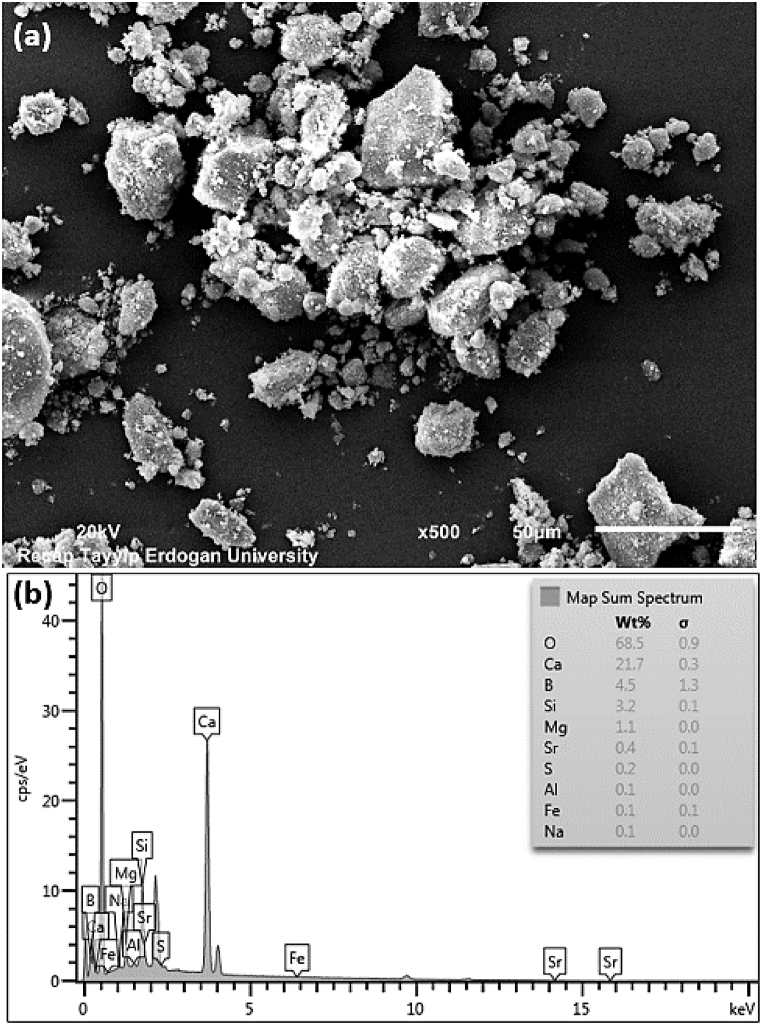
(a) SEM image under × 500 and (b) EDS spectrum of natural colemanite.

Microstructure images of the NC mineral were taken via a stereo optical microscope (SOM) (Olympus, model 'SZ61') and an SEM (Jeol, model 'JSM-6610′). Fig. 1(a) and (b) show SOM images of NC mineral under x6 and x45 magnification, respectively. In Fig. 1(a), it appears that the color of the NC mineral is gray; that is, it is a single color. However, small amounts of black, orange, and bright white colors are also seen in the SOM image under x45 magnification (Fig. 1(b)). These colors refer to the presence of other minerals in the NC. Yildiz [16] reported in a study that such colemanite mineral is not pure and contains impurities like calcite, clay minerals, and arsenic. In addition, it was determined that the colemanite was agglomerated in some places and its average particle size was below 50 μm. In order to make a more detailed analysis in powder technology, the SEM image of the NC mineral at x500 is illustrated in Fig. 2(a). While the particle shape of the NC mineral is usually angular/irregular, the shape of some particles is rounded/spherical. The size of the particles ranges from roughly 50 μm to <1 μm, thus providing an approximately homogeneous distribution at the micron scale.

The microchemical elemental measurement of the NC mineral was investigated with an energy-dispersive X-ray spectrometer (EDS) (Oxford Inst., model 'x-act') incorporated into the SEM. Weight % data were determined using region mapping scanning. Fig. 2(b) depicts the EDS spectrum and elemental analysis values of the NC mineral. The maximum 3 peaks within the spectrum were oxygen (O), calcium (Ca), and silicon (Si), respectively. In addition, magnesium (Mg), boron (B), strontium (Sr), sulfur (S), aluminum (Al), iron (Fe), and sodium (Na) peaks were also detected. When the values of the elements were examined, the three highest values were measured as O, Ca, and B; thus, their total percentage values were calculated as 94.7. Three interpretations can be made from these data. First, considering formula 2CaO ·3B_2_O_3_·5H_2_O, the NC mineral is not a pure colemanite. Secondly, there are other minerals in small amounts (5.3 %) in the colemanite mineral. Finally, the reason for the absence of B among the three highest peaks in the spectrum is that it is difficult to detect because its atomic number is less than 11 [23]. These results explain very well the different colors detected in the SOM image in Fig. 1(b) and are moreover in good agreement with the chemical composition measurement in Table 1.

#### 2.1.2 Calcination process

The purpose of calcining the NC mineral is to evaporate 5 mol of crystal water in the colemanite mineral (2CaO·3B_2_O_3_·5H_2_O) and thus to increase the percentage of B_2_O_3_. The next process is to add the calcined colemanite mineral to the concrete and examine its effect on the concrete.

**Fig. 3:**
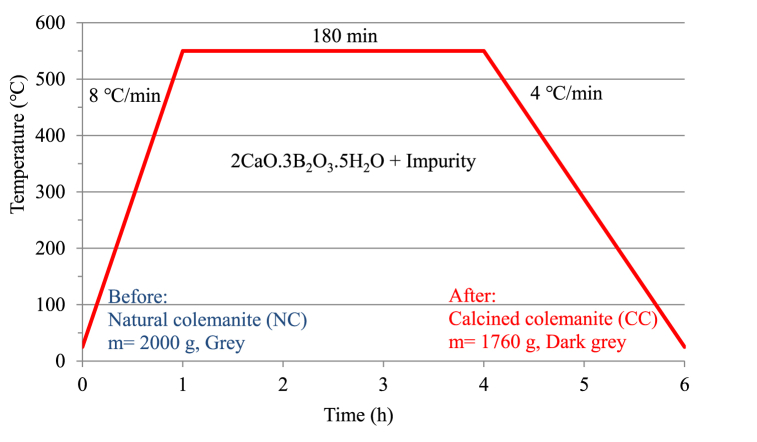
Diagram of calcination process for natural colemanite.

According to the literature search [16,[24], [25], [26], [27]], the program setting was made by placing the NC mineral in the muffle furnace (Nabertherm, model 'N50'). Afterward, the furnace reached to 550 °C in 1 h and was kept there for 3 h. For simplicity, calcined colemanite was labeled CC. Fig. 3 shows the calcination process applied to the NC mineral.

2000 g of natural colemanite mineral was put into the furnace, but after the calcination process, the colemanite mineral was weighted as 1760 g (Radwag, model 'WLC 20/A2', Sensitivity d = 0.1 g). In other words, there was a mass loss of approximately 12 %.

#### 2.1.3 Physical property tests

The crystal structure of NC and CC minerals was examined by X-ray diffractometer (XRD, Rigaku, model 'SmartLab'). The operation conditions were set as voltage 40 kV, current 30 mA, scanning speed 5°/min, and scanning step 0.02°. The CuK_α_ radiation wavelength (λ) was 1.541 Å. After the measurement, qualitative analysis was performed via the device's PDXL software.

The thermal property of the NC and CC minerals was analyzed by the ThermoGravimetry/Differential Thermal Analysis (TG/DTA) (Netszch, model 'STA 449 F3 Jupiter') instrument. Tests were carried out at a temperature range of 25−1000 °C, heating ratio of 10 °C/min, and dry air (%20 N_2_−%80 O_2_) atmosphere.

The amount of precious B_2_O_3_ in the colemanite mineral was determined using the titrimetric method. The measurement was made in TS EN ISO/IEC 17025 standard.

### 2.2 Preparation and characterization of concrete samples

#### 2.2.1 Materials and mixture design

**Fig. 4:**
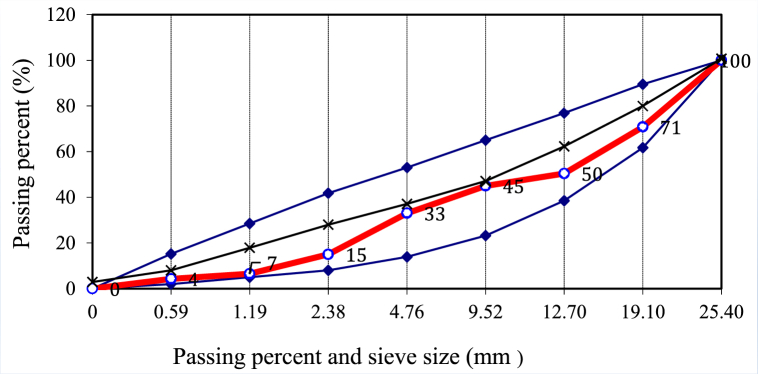
Granulometry curve of aggregates.

Portland cement (CEM I 42.5 R) and crushed aggregate with a maximum size of 25.4 mm were utilized in the concrete production. The amount of aggregate was determined from the granulometry curve shown in Fig. 4. Basalt-containing aggregate was used as the aggregate type. Particle density and water absorption tests were performed according to EN 1097–6:2013 standard [28]. Based on the EN 12620 concrete aggregate standard, lightweight aggregates should be lighter than 2.4 g/cm^3^, and normal weight aggregates should be 2.4−2.8 g/cm^3^. Heavy aggregates are heavier than 2.8 g/cm^3^. This specification criterion and the fact that the water absorption rate of concrete aggregates should be less than 3 % by mass according to EN 12620 [29] were taken into consideration. While preparing the concrete, the mixture calculation was made according to the ASTM C94/C94M − 20 [30] standard. Potable tap water was utilized for the preparation of fresh concrete. For present study, the w/c ratio was found to be 0.40.

**Fig. 5:**
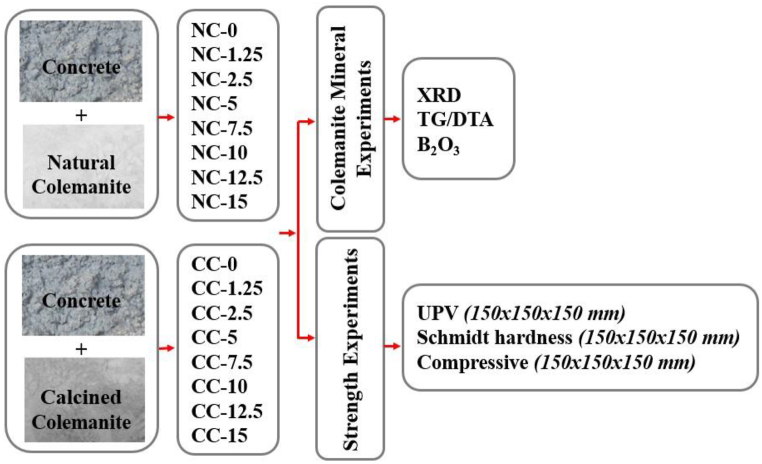
Flowchart for the experimental method.

Fig. 5 illustrates the flowchart of the experiments. Both NC and CC minerals were added in 7 different ratios (1.25 %, 2.5 %, 5 %, 7.5 %, 10 %, 12.5 %, and 15 %) by cement weight to the mixture calculation based on material densities. Considering these rates of increase, the mixtures were labeled as given in Table 2. Also, material quantities are listed in Table 2.

**Table 2:** The rates of components in 1 m^3^ of concrete mixture for natural and calcined colemanite minerals.

Samples		Cement(kg)	Water (l)	Water/Cement	0–4 mm (kg)	4–16 mm (kg)	Natural/Calcined colemanite (kg)
Ref	Ref	370	148	0.4	788	1020	0
NC1.25	CC1.25	370	148	0.4	788	1020	0.462
NC2.5	CC2.5	370	148	0.4	788	1020	0.925
NC5	CC5	370	148	0.4	788	1020	1.850
NC7.5	CC7.5	370	148	0.4	788	1020	2.775
NC10	CC10	370	148	0.4	788	1020	3.700
NC12.5	CC12.5	370	148	0.4	788	1020	4.625
NC15	CC15	370	148	0.4	788	1020	5.550

In this study, the densities of concrete and colemanite (NC) are 2.4 g/cm^3^ and 2.42 g/cm^3^, respectively. C25/30 grade concrete mixtures with an initial slump of about 100 ± 10 mm were produced. Fresh concrete was filled into cube molds (15 cm × 15 cm × 15 cm), and then a test vibrator was used to ensure that there was no space inside. The poured samples were maintained in the curing pool in a humid laboratory condition under an ambient temperature of 22 ± 3 °C for 24 h.

The poured samples were cured for 28 days in accordance with ASTM C192/C192M − 19 [31]. However, the samples with more than 5 % CC mineral additive were observed to disperse approximately 1 h after being placed in the curing pool. Afterward, the cured samples were allowed to dry for 24 h under normal air conditions.

#### 2.2.2 UPV, Schmidt hammer, compressive strength tests

Ultrasonic pulse velocity measurement is performed with a special electronic device. The wave velocity is calculated by measuring the time taken from one surface of the cube samples to the other surface of the longitudinal and transverse ultrasonic waves. The UPV measurement was conducted using a handy Proceq Pundit Lab 200 device with an accuracy of ±0.2 V in compliance with ASTM C597 [32]. Thanks to the UPV, relationships between the compressive strength and other properties of the concrete can be roughly determined. The presence of cracks and voids in the concrete structure indicates a lower UPV, while homogeneous and quality concrete implies a higher UPV [33].

Schmidt hammer hardness is a non-destructive measurement used to identify the approximate value of the uniaxial compressive strength of the material. The Schmidt hammer is faster and cheaper test than the others, but the accuracy of the measurement is on average 10 % lower. The testing principle is to press the surface of the test body onto the hammer. The test hammer, which is kept fixed parallel to the sample to apply full load, is pressed against the sample, and measurements are taken from 5 different points at right angles to the sample. Depending on the hardness of the sample, data between 0 and 100 are obtained. The body hardness is eliminated from the indicator. Subsequent to the impact, the measured data indicator has to be fixed. The hardness test is related to the degree of rebound of the hammer from the surface [34,35]. The surface hardness test was carried out using a UTEST N-type Schmidt hammer with an accuracy of ±0.3 according to ASTM C805 [36].

Concrete compressive strength is measured with uniaxial strength, which is the method that gives the most accurate results. For this study, the cubic sample size was 15 cm × 15 cm × 15 cm. The smooth surfaces of the sample were placed between two plates of a UTEST Multiplex electromechanical testing machine, which has an accuracy of ±0.6 MPa. The test was performed at loading rates of 3 kN/s and 1 mm/min until the sample broke. All samples were subjected to the uniaxial compression test in ASTM C39/C39M − 14 [37].

## 3 Results and discussion

### 3.1 Properties of calcined colemanite mineral

#### 3.1.1 Crystal structure analysis

**Fig. 6:**
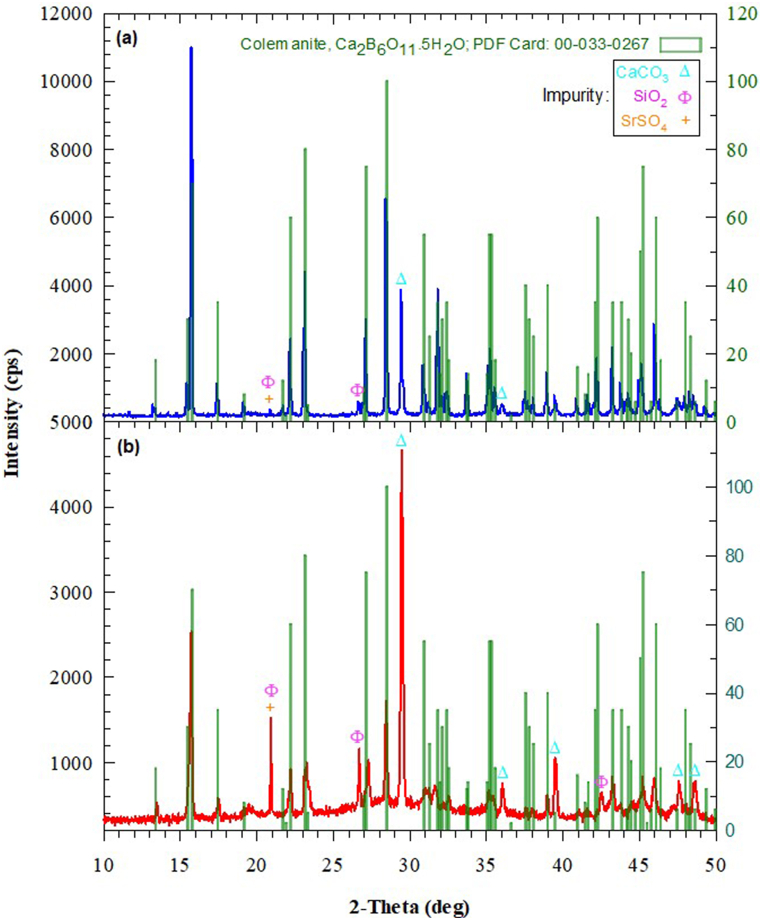
XRD patterns of (a) natural colemanite and (b) calcined colemanite.

Fig. 6(a) displays the XRD diffractogram of the natural colemanite mineral. The three highest intensity peaks of the NC mineral are *I* = 11000 (cps) for *2θ* = 15.78°, *I* = 6580 (cps) for *2θ* = 28.48°, and *I* = 4420 (cps) for *2θ* = 23.12°, in turn. Fig. 6(b) shows the XRD diffractogram of the calcined colemanite mineral. The three highest intensity peaks of the CC mineral are *I* = 4660 (cps) for *2θ* = 29.48°, *I* = 2530 (cps) for *2θ* = 15.72°, and *I* = 1720 (cps) for *2θ* = 28.44°, respectively. It is obvious that the crystal structure changes with the calcination process and this can be expressed with a few comments. First, there has been a decline in the number of peaks, which probably means that crystal water has been removed from the structure. Second, the intensity of the highest peak of the NC mineral has decreased by 77 % from 11000 (cps) to 2530 (cps). Third, the intensity of the highest peak of the CC mineral has heightened by 20 % from 3890 (cps) to 4660 (cps). Fourth, the peak number of impurity mineral(s) has increased from 4 to 6. Fifth, the dominance of the impurity mineral(s) has enhanced.

In the qualitative analysis performed on the XRD device, it was found that the NC mineral mostly matched with the colemanite file (monoclinic, space group: *P*2_1_/*a*, *d*_*meas*_ = 2.42) with PDF card no:00-033-0267. Besides, the highest intensity peak of NC mineral corresponded to the Miller index of (020). This indicates that the major phase is a colemanite mineral. However, the presence of impurity minerals (minor phase) such as calcite (CaCO_3_), silica (SiO_2_), celestine (SrSO_4_) [16,38] was also detected in the NC mineral. This result confirms the results of the chemical composition in Table 1, the SOM image in Fig. 1(b), and the EDS spectrum in Fig. 2(b).

Similarly, in the qualitative analysis, it was observed that the CC mineral matched the colemanite file, but both the peak intensity and the peak number of the impurity mineral(s) were higher. It was determined that the highest peak of the CC mineral coincided with the calcite (CaCO_3_, syn) file with PDF card no: 00-005-0586, and furthermore corresponded to the Miller index of (104). This indicates that the major phase is impurity minerals. At the same time, the presence of impurity phases such as silica and celestine was also found in the CC mineral composition.

Kutuk [18] reported that the highest peak intensity at *2θ* = 15.8° of ground colemanite mineral (natural, −45 μm) belonged to pure colemanite mineral, and also the peak at *2θ* = 29.5° pertained to the impurity mineral. Yildiz [16] declared that the calcined colemanite mineral (−250 μm, B_2_O_3_: 42 wt%) at 600 °C decomposed into amorphous forms of B_2_O_3_ and CaO, components such as CaCO_3_ and SrSO_4_ did not dissolve, and the highest intensity peak at *2θ* ≈ 29.5° belonged to the CaCO_3_. Akpinar et al. [39] published that the XRD diffractogram of ground colemanite (natural, −75 μm, conventional method) at 450 °C hardly changed, whereas the XRD diffractogram at 700 °C changed drastically. They stated that the crystal structure at 700 °C turned into amorphous calcium borate (Ca_3_(BO_3_)_2_) form, and that the highest peak at *2θ* ≈ 29° also pertained to calcite. As a result, previous studies are consistent with the results of this study.

Briefly, in present study, at the end of the calcination process at 550 °C, it was understood that some crystal water in the colemanite mineral decomposed; that is, the crystalline colemanite mineral did not transform into a completely amorphous structure or a new crystal structure.

#### 3.1.2 Thermal analysis

**Fig. 7:**
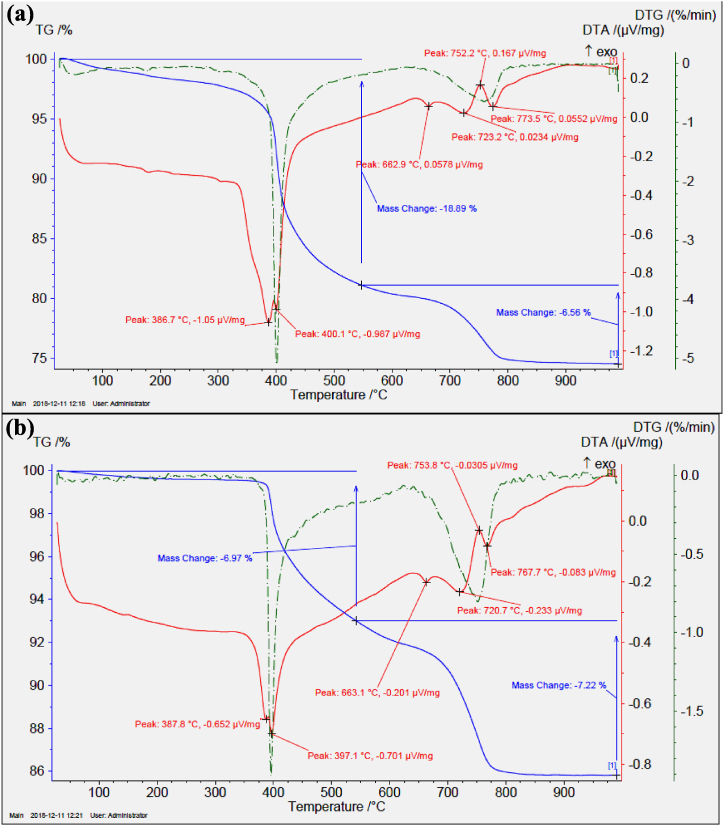
TG/DTA curves of (a) natural colemanite and (b) calcined colemanite.

TG/DTA curves for NC mineral in Fig. 7(a) and for CC mineral in Fig. 7(b) are shown. The TG (mass change) value of NC mineral descended to 98 % at 300 °C, 97 % at 350 °C, 92 % at 400 °C, 84.5 % at 450 °C, 82 % at 500 °C, 81 % at 550 °C, and 80.5 % at 600 °C. At the same time, a high intensity narrow peak was observed at ∼400 °C in the DTG (Derivative TG) curve of the NC mineral. Similarly, the TG value of CC mineral decreased to 99.5 % at 300 °C, 99.5 % at 350 °C, 97.5 % at 400 °C, 95 % at 450 °C, 94 % at 500 °C, 93 % at 550 °C, and 92 % at 600 °C. In addition, a narrow peak with high intensity appeared in the DTG curve of CC mineral at ∼397 °C. From the TG data, it was noticed that the NC mineral reached saturation at 550 °C (for step 1), but there was still a 7 % loss for the CC mineral at the same temperature. This means that there was still a small amount of crystal water in the colemanite. From the DTG data, it was decided that there was a small decrement of 3 °C with the calcination process.

When the DTA curve was examined, endothermic twin peaks with high intensity were observed at 387 °C and 400 °C for the NC mineral and also at 388 °C and 397 °C for the CC mineral. The twin peaks here are the characteristic peaks of the colemanite mineral [25]. More clearly, a great decomposition appeared at 400 °C. The reason for this is the breaking of OH bonds in the crystal structure (2CaO·3B_2_O_3_·5H_2_O) or the removal of partial mole amount of crystal H_2_O [26].

It is known that the mass loss between 300 °C and 600 °C is approximately 18 %, which corresponds to 5 mol of crystal water (dehydroxylation and dehydration) [38]. However, in the weighing process at the end of the calcination process in present study, the colemanite mineral decreased from 2000 g to 1760 g, in other words, there was a 12 % mass loss. The mass loss at 550 °C is 19 % (including 2 % moisture) in Fig. 7(a) for NC mineral and 7 % (including 0.5 % moisture) in Fig. 7(b) for CC mineral. The difference (12 %) between these two percentages fully coincides with the weight percentage. Therefore, the following calculation can be made: Approximately 71 %, or 3.5 mol of crystal water has been released from the structure. This situation can be roughly expressed by Eq. (1):

(1) $$2\text{CaO}\cdot 3B_{2}O_{3}\cdot 5H_{2}O\;\to 2\text{CaO}\cdot 3B_{2}O_{3}\cdot 3/2H_{2}O+{\left({7/2H}_{2}O\right)}_{vapor}$$

Akpinar et al. [39] declared that the mass loss of 12.14 % for ground colemanite mineral (natural, −75 μm) between 344 °C and 440 °C is significantly smaller than the theoretical values, and such a loss arises from the calcite mineral. The fact that the major phase detected in the XRD pattern in Fig. 6(b) in this study is calcite confirms the interpretation of Akpinar et al. [39]. In addition, briefly expressed for the DTA curve of the NC mineral, the decomposition of calcite mineral given in Eq. (2) occurred at 663 °C and 723 °C, the release of CO_2_ happened at 752 °C, and the recrystallization of anhydrous amorphous colemanite phase (Ca_2_B_6_O_11_) given in Eqs. (3), (4) occurred at 774 °C [38,40].

(2) $$\text{CaC}O_{3}\to \text{CaO}+{\left(CO_{2}\right)}_{vapor}$$

(3) $$2\text{CaO}\cdot 3B_{2}O_{3}:\text{fully}\;\text{anhydrous}\;\text{amorphous}\;\text{colemanite}$$

(4) $$2\text{CaO}\cdot 3B_{2}O_{3}\cdot 3/2H_{2}O:\text{mostly}\;\text{anhydrous}\;\text{amorphous}\;\text{colemanite}\;\text{for}\;\text{this}\;\text{study}$$

In short, it is possible to say that a large amount of crystal water in the colemanite mineral was removed from the structure owing to the calcination process carried out at 550 °C for this study.

#### 3.1.3 B_2_O_3_ component analysis

B_2_O_3_ component values of NC and CC minerals are given in Table 3. While the B_2_O_3_ weight percentage was 40.30 for the NC mineral, it was 44.62 for the CC mineral. Thus, it was concluded that the amount of B_2_O_3_ improved by 11 % as a result of the calcination process. This result is very close to the 12 % mass loss value calculated from the TG curves in Fig. 7.

**Table 3:** Chemical composition of B_2_O_3_ amount for the colemanite minerals.

Minerals	B_2_O_3_ (wt.%)	
	Measured product	Commercial (Catalog) product
Natural colemanite	40.30 ± 0.50	40.00 ± 0.50
Calcined colemanite	44.62 ± 0.50	null

Gulensoy and Kocakerim [24] identified that in the calcination curve of the colemanite mineral (B_2_O_3_: 50.81 wt%), all crystal water dissolved at 550 °C, not only the B_2_O_3_ value but also the CaO value increased, and moreover, the B_2_O_3_/CaO ratio did not change. Based on these findings, it was realized that the B_2_O_3_/CaO ratio in this study was 1.49 and at the same time the CaO ratio increased. This inference is consistent with the determination of calcite as the major phase detected in the XRD pattern in Fig. 6(b).

### 3.2 Properties of concrete samples with calcined colemanite additive

#### 3.2.1 Ultrasonic pulse velocity analysis

**Fig. 8:**
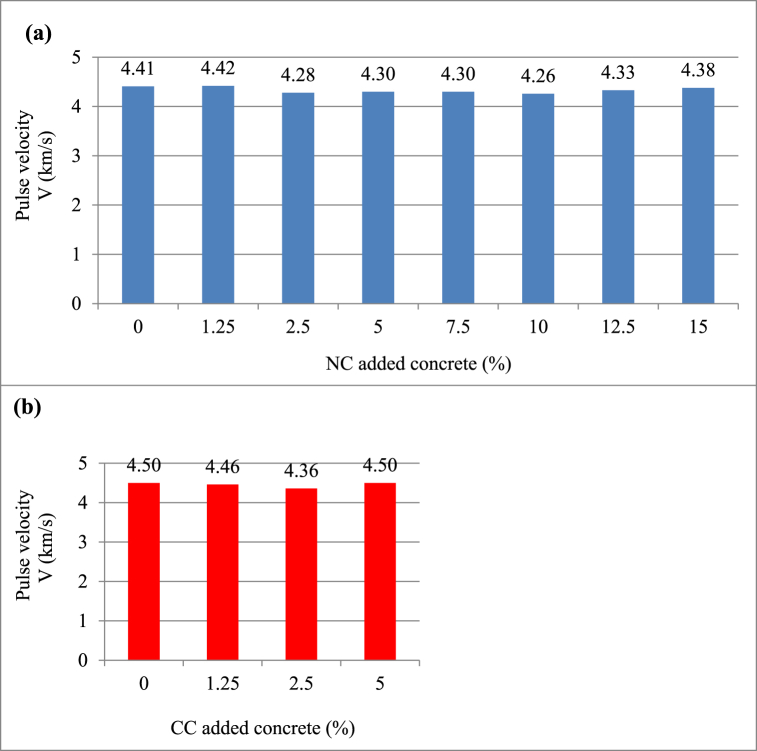
Ultrasonic pulse velocity values of (a) natural colemanite and (b) calcined colemanite added concrete samples.

Fig. 8(a) and (b) show the 28-day UPV results of concrete samples with NC and CC additives, respectively. It is well-known that as the number of voids in the concrete diminishes, compactness rises; therefore, the velocity increases and the strength improves. It was understood that the UPV values did not change roughly with the increase in both the NC ratio and the CC ratio, so there was no linear relationship between them.

The classification made by Whitehurst [41] is listed in Table 4, and also the assessment of the findings of this study is presented in Table 5. When the UPV results were examined, it was deduced that the addition of colemanite to the concrete did not substantially change in the transition velocity, that all samples were in good class, and that the void structure of the samples was close to each other. In this sense, it is a very good situation that the void structure is close to each other.

**Table 4:** Practical evaluation of ultrasonic pulse velocity values (Whitehurst, 1951).

Ultrasonic pulse velocity (km/s)	Concrete quality
>4.5	Very good
3.5–4.5	Good
3.0–3.5	Middle
2.0–3.0	Weak
<2.0	Too weak

**Table 5:** Practical evaluation of ultrasonic pulse velocity results for natural and calcined colemanites.

Samples		Ultrasonic pulse velocity (km/s)	
		For NC	For CC
Ref	Ref	Good	Good
NC1.25	CC1.25	Good	Good
NC2.5	CC2.5	Good	Good
NC5	CC5	Good	Good
NC7.5	CC7.5	Good	null
NC10	CC10	Good	null
NC12.5	CC12.5	Good	null
NC15	CC15	Good	null

In a study by Gencel et al. [42], the addition of colemanite (natural, B_2_O_3_: 39.48 wt%) to concrete at different rates did not make a remarkable difference in UPV; what's more, the concrete grade was moderate and good in the evaluation made according to Whitehurst [41]. Celik et al. [43] found that the UPV results of the mixtures obtained by adding different rates of ground colemanite mineral (natural, B_2_O_3_: 40 wt%) and synthetic fibers to the concrete were almost the same. This is an indication that the added materials didn't impact the uniformity.

#### 3.2.2 Schmidt hardness analysis

**Fig. 9:**
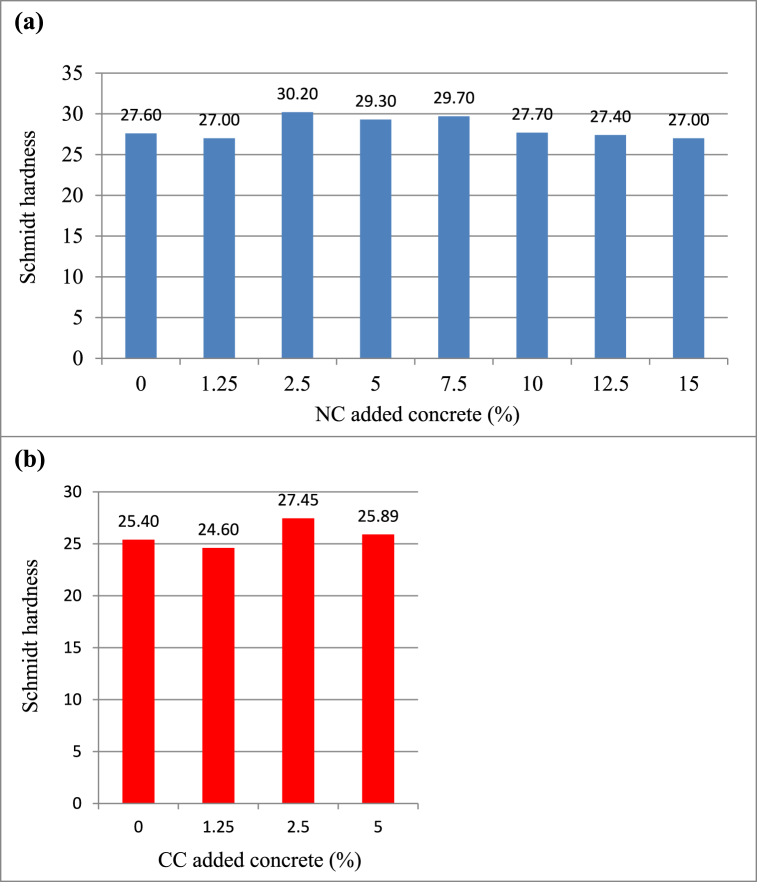
Schmidt Hammer values of (a) natural colemanite and (b) calcined colemanite added concrete samples.

Fig. 9(a) displays the 28-day surface hardness results of the samples with NC additives. The sample with the highest surface hardness is NC2.5 with a 9 % increment in comparison with the reference sample, whereas the samples with the lowest surface hardness are NC1.25 and NC15. From this result, it was realized that there was no systematic relationship between NC additive ratio and surface hardness. One reason for this may be the small number of samples. As can be seen in Table 6, Fayetorbay [44] stated that no relationship was found between compressive strength and surface hardness in the experiments performed with 3 different types of cement. In addition, other studies reveal that the technical properties of the sample vary with respect to thickness [27,45]. The surface hardness value represents the 30−50 mm outer layer of the thickness of the concrete; consequently, it is used to estimate the approximate compressive strength [46]. Yet, the compressive strength value indicates the entire layer of concrete. It is inferred from this that taking the entire thickness into account for surface hardness in concrete may not always give accurate results. More clearly, the Schmidt hammer test on NC added concrete samples in this study did not reflect the truth at early ages.

**Table 6:** 28 days Schmidt hammer and compressive strength values of concretes given by Fayetorbay (2013).

	Cement type	No	Schmidt hammer	Compressive strength (MPa)
(28 days)	CEM I 42.5 R	1	29.5	49.99
		2	30.0	47.40
		3	27.7	50.13
	CEM II 32.5 B-S	1	21.3	38.05
		2	18.8	36.20
		3	19.8	35.79
	Boron active belite cement	1	21.2	35.28
		2	20.9	35.59
		3	22.4	36.05

In Fig. 9(b), the 28-day surface hardness results of samples with CC additives are depicted. The sample with the biggest surface hardness is CC2.5 with an increase of 8 % according to the reference sample, while the sample with the smallest is CC1.25. Kutuk et al. [47] substituted the natural colemanite mineral (−75 μm, B_2_O_3_: 40 wt%) to cement at 1 %, 2 %, 3 %, 4 %, and 5 % ratios. Concrete samples’ abrasion resistance was assessed using the Bohme experiment, so the correlation between experiment findings and the Schmidt hammer was investigated. They said that the substituted colemanite enlarged the abrasion amounts in 7-day and 28-day concrete samples, but up to 3 % substituted colemanite decreased the abrasion amount with ascending hardening age (90 days). In other words, this 3 % substitution value is similar to the sample with CC2.5 (2.5 %) additive.

#### 3.2.3 Compressive strength analysis

**Fig. 10:**
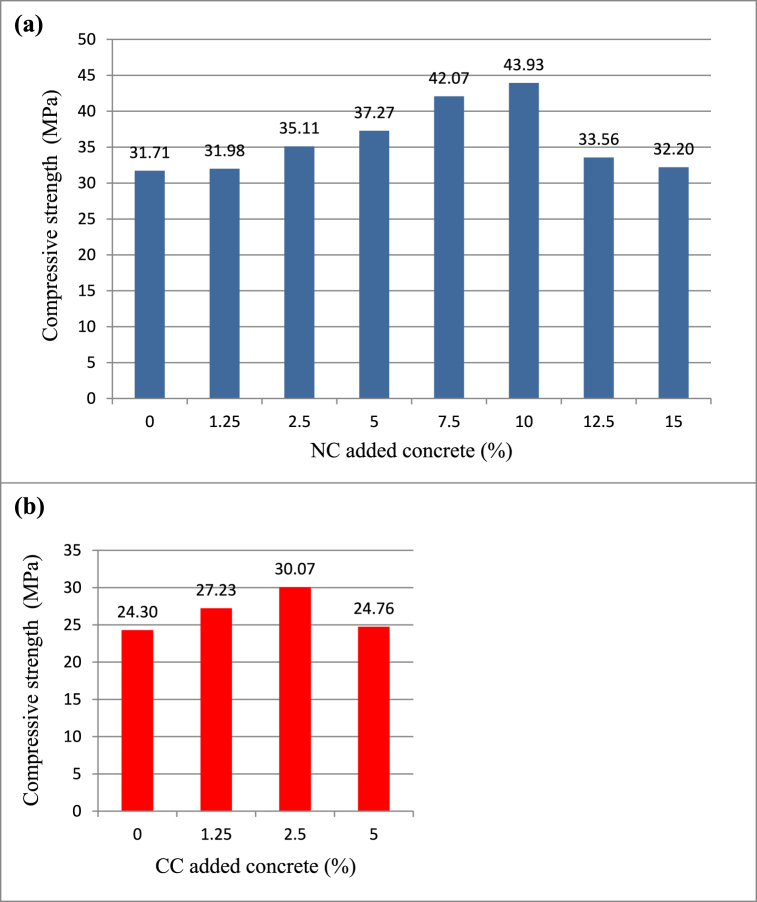
28 days compressive strength values of (a) natural colemanite and (b) calcined colemanite added concrete samples.

28-day compressive strength values of samples with NC additives are given in Fig. 10(a). The lowest compressive strength belongs to the reference sample with 31.71 MPa, while the biggest compressive strength belongs to the NC10 sample with 43.93 MPa. That is, the compressive strength improved by 39 % and gradually increased up to 10 % additive. It is believed that the factor contributing to the compressive strength is the B_2_O_3_ content in the colemanite mineral.

Kara et al. [48] replaced the natural colemanite mineral (−75 μm, B_2_O_3_: 40 wt%) with cement by weight in the concrete mixture and deduced that up to 4 % colemanite substitution increased the compressive strength. In addition, Kutuk-Sert [17] added the natural colemanite mineral (−45 μm, B_2_O_3_: 40 wt%) to the concrete sample and found that the NC mineral enhanced the compressive strength by 9 %. These studies suggest that the colemanite mineral has a positive advantage on concrete.

Celik et al. [43] added 10 %, 20 %, 30 %, and 40 % natural colemanite minerals (B_2_O_3_: 40 wt%) to metakaolin added concrete mixtures and found a 2 % improvement in compressive strength at only a 10 % ratio. Such a 10 % ratio is consistent with the NC10 (10 %) sample in this study.

Aksogan et al. [49] declared that concrete samples with 0.5 % and 1 % natural colemanite (−75 μm, B_2_O_3_: 40 wt%) additives had successively 2.8 % and 9.6 % larger compressive strength than the control sample. Such a 9.6 % improvement is in agreement with the 11 % improvement obtained for the NC2.5 (2.5 %) sample. In addition, Kula et al. [50] substituted 1 % and 3 % colemanite waste (−90 μm, B_2_O_3_: 17.65 wt%) in Portland cement mixtures and subsequently obtained 8.8 % and 3 % higher compressive strength, in turn, than the Ref(C41.8) sample. Even so, in this study, the increase in strength is 1 % for the NC1.25 sample and 10 % for the NC2.5 sample. The cause of this disparity is assumed to be the cement mix, higher strength reference sample, coarser particle size, lower B_2_O_3_ content, and bigger impurity component.

Ozturk et al. [51] ascertained approximately 15 % greater compressive strength in concrete samples with 5 % borogypsum (B_2_O_3_: 1.05 wt%) than the sample with Portland cement. Additionally, the concrete sample substituted with 5 % natural colemanite mineral (B_2_O_3_: 40.51 wt%) was found to have 23 % higher compressive strength than the concrete sample containing Portland cement. This 23 % increment roughly confirms the 18 % increment gained in the concrete sample with 5 % NC in this study.

Fig. 10(b) shows the 28-day compressive strength values of samples with CC additives. The compressive strength is 24.3 MPa for the reference sample, whereas it is 30.07 MPa with an increase of 24 % for the CC2.5 sample. It was concluded that the compressive strength of the concrete sample improved remarkably thanks to the addition of both NC mineral and CC mineral.

The compressive strength of concrete samples with NC and CC additives were deduced to decrease after a specific ratio. Moreover, it was observed that the setting stopped at the ratio of 5 % in CC mineral. This negative result is attributed to the B_2_O_3_ content, which retards the setting time of concrete [52]. However, it was noticed that higher compressive strength could be achieved at lower rates for samples with CC mineral. This positive result may be due to the 11 % increase in the amount of B_2_O_3_ (see Table 3) as a result of the calcination process.

In the light of the XRD data in Fig. 6 and TG/DTA data in Fig. 7, the colemanite mineral lost most of its crystal water in consequence of the calcination process. The excess in the amount of B_2_O_3_ given in Table 3 is probably due to the reduction in the amount of crystal water. Therefore, it was inferred that the compressive strength of the CC mineral declined after the additive value of 2.5 % because the sensitivity of the CC mineral to water increased.

**Fig. 11:**
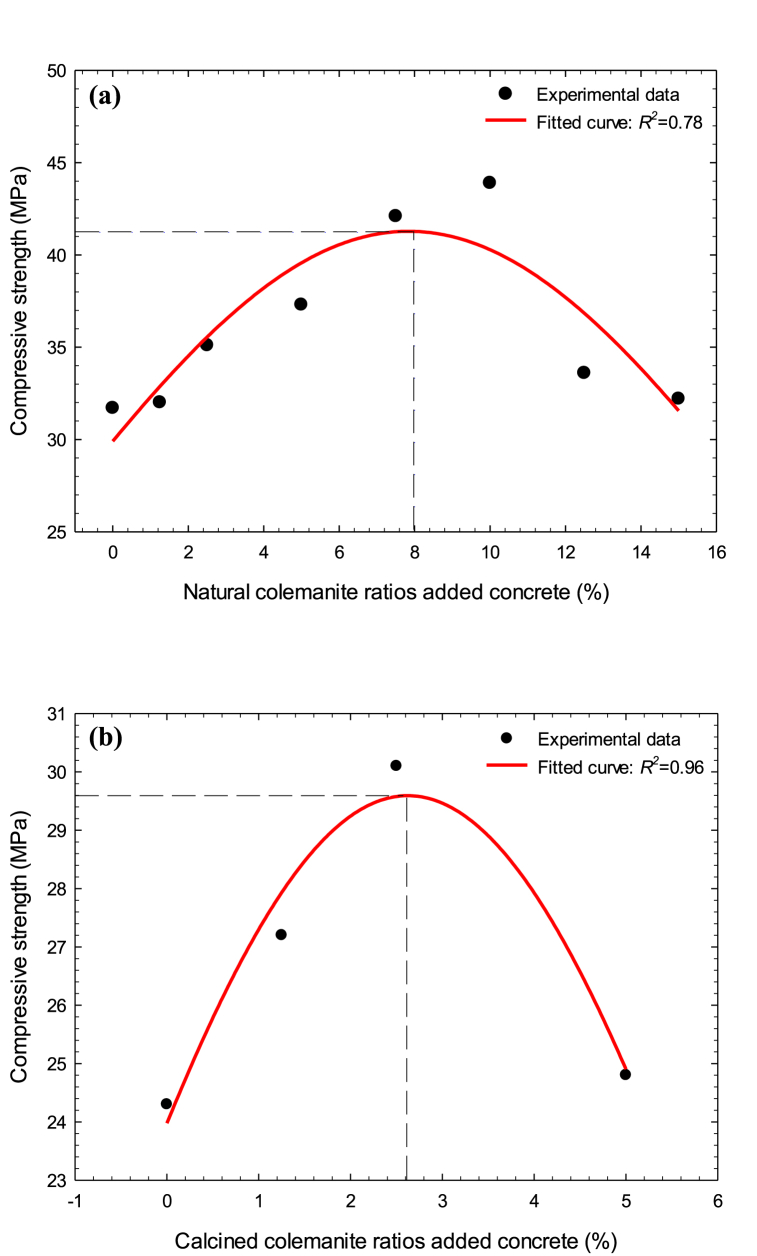
Fitted curves of (a) natural colemanite and (b) calcined colemanite added concrete samples.

The fitted curves of the samples with NC and CC additives are displayed in Fig. 11. For experimental data, the *R*^*2*^ (coefficient of determination) value was found to be 0.78, that is, it has a strong correlation (Fig. 11(a)). Taking into account the fitted curve, the optimal colemanite ratio is approximately 8.0 %, and the corresponding compressive strength is 41.3 MPa. According to this finding, the compressive strength increased by 30 % above the reference sample. Similarly, as seen in Fig. 11(b), the *R*^*2*^ value was calculated as 0.96, indicating a very strong correlation. When the fitted curve is taken into consideration, the optimal calcined colemanite ratio is approximately 2.6 %, and the corresponding compressive strength is 29.6 MPa. In other words, the compressive strength improved by 22 % in comparison with the reference sample.

In the last review study [53], it has been underlined that utilizing colemanite mineral as filler is suitable and this will be a trend in the concrete sector in the future, especially due to its neutron shielding property. In addition, in this study, considering the analysis in Fig. 11, it is suggested to add colemanite mineral to C25 concrete grade, not C30 concrete grade, since a 20−25 % improvement in compressive strength was determined. For example, C25 concrete [54], which is the most widely used concrete grade with 43.70 % as the application in Turkey, will be upgraded to concrete grade in the range of C30−35 with the addition of colemanite. In this way, cement will be saved and environmental pollution will be reduced.

## 4 Conclusion

In the present study, the role of calcined colemanite mineral on the mechanical properties of concrete was investigated. In the initial step of the experimental procedure, natural colemanite mineral was subjected to a calcination process. In the other step, both NC and CC minerals were added to the concrete in different ratios (1.25 %, 2.5 %, 5 %, 7.5 %, 10 %, 12.5 %, and 15 %). Notable results are given below.

• XRD analysis showed that upon the completion of the calcination process, the crystal structure of the NC mineral changed and the major phase became the impurity phase calcite.
• TG/DTA analysis revealed that about 71 %, or 3.5 mol, of crystal water in CC mineral was removed from the structure.
• The percentage of B_2_O_3_ component increased by 11 % with the calcination process. This value is in good agreement not only with the 12 % mass loss value calculated from the TG curves, but also with the 12 % loss value measured from the balance weighing.
• The compressive strength was found to be high for all colemanite-added concrete samples. In the sample with a 10 % NC additive, the compressive strength increased by about 39 %. Similarly, in the sample with a 2.5 % CC additive, the compressive strength improved by approximately 24 %. Besides, it was concluded that high compressive strength could be achieved with a low additive ratio because of the calcination process.
• It was determined that CC mineral delayed the setting time of the concrete after a 2.5 % additive rate and even stopped it after a 5 % additive rate. Due to the fact that the B_2_O_3_ component heightened by 11 % and the amount of crystal water diminished by 71 % at the same time, it was thought that the sensitivity of the CC mineral to water increased, and therefore the setting time was negatively affected.

It is strongly recommended to use the colemanite mineral additive in cement and concrete for future studies in order to evaluate the colemanite mineral reserve, save cement, and improve strength. Additionally, density measurements of NC and CC minerals should be taken and their microstructures analyzed. Moreover, the colemanite additive will reduce both the dependency on cement and CO_2_ emissions and thus, important steps will be taken for the environment.

## CRediT authorship contribution statement

**Nihat Utku Guner:** Writing – original draft, Visualization, Validation, Resources, Methodology, Investigation, Data curation. **Sezai Kutuk:** Writing – review & editing, Writing – original draft, Visualization, Validation, Resources, Methodology, Investigation, Funding acquisition, Formal analysis, Data curation, Conceptualization. **Tuba Kutuk-Sert:** Writing – review & editing, Writing – original draft, Supervision, Conceptualization.

## Data and code availability statement

Data will be made available on request.

## Ethics statement

This study did not involve human or animal subjects, and thus, no ethical approval was required.

## Funding statement (Open access)

This study has been supported by the Recep Tayyip Erdoğan University Development Foundation (grant number: 02024007019015).

## Declaration of competing interest

The authors declare that they have no known competing financial interests or personal relationships that could have appeared to influence the work reported in this paper.
